# Analysis of the Molecular Mechanisms of Reepithelialization in *Drosophila* Embryos

**DOI:** 10.1089/wound.2014.0549

**Published:** 2016-06-01

**Authors:** Yutaka Matsubayashi, Tom H. Millard

**Affiliations:** Faculty of Life Sciences, The Healing Foundation Centre, University of Manchester, Manchester, United Kingdom.

## Abstract

**Significance:** The epidermis provides the main barrier function of skin, and therefore its repair following wounding is an essential component of wound healing. Repair of the epidermis, also known as reepithelialization, occurs by collective migration of epithelial cells from around the wound edge across the wound until the advancing edges meet and fuse. Therapeutic manipulation of this process could potentially be used to accelerate wound healing.

**Recent Advances:** It is difficult to analyze the cellular and molecular mechanisms of reepithelialization in human tissue, so a variety of model organisms have been used to improve our understanding of the process. One model system that has been especially useful is the embryo of the fruit fly *Drosophila*, which provides a simple, accessible model of the epidermis and can be manipulated genetically, allowing detailed analysis of reepithelialization at the molecular level. This review will highlight the key insights that have been gained from studying reepithelialization in *Drosophila* embryos.

**Critical Issues:** Slow reepithelialization increases the risk of wounds becoming infected and ulcerous; therefore, the development of therapies to accelerate or enhance the process would be a great clinical advance. Improving our understanding of the molecular mechanisms that underlie reepithelialization will help in the development of such therapies.

**Future Directions:** Research in *Drosophila* embryos has identified a variety of genes and proteins involved in triggering and driving reepithelialization, many of which are conserved in humans. These novel reepithelialization proteins are potential therapeutic targets and therefore findings obtained in *Drosophila* may ultimately lead to significant clinical advances.

**Figure:**
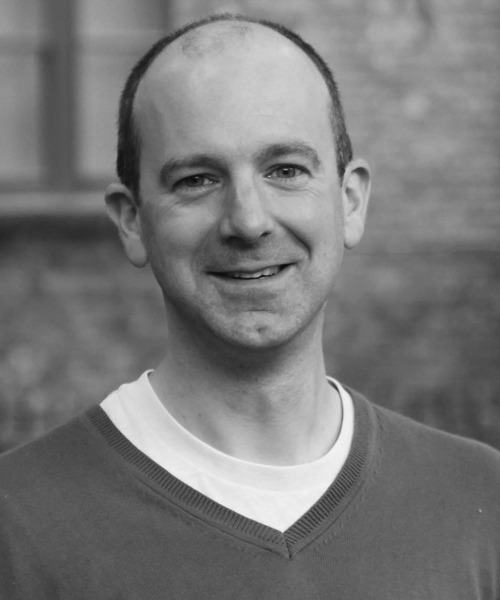
**Tom H. Millard, PhD**

## Scope and Significance

Repair of the epidermis, or reepithelialization, is a key event during wound healing. The *Drosophila melanogaster* embryo has proved to be a useful model system for analyzing the fundamental cellular and molecular mechanisms that underlie the process. This review will discuss the insights gained from studying reepithelialization in *Drosophila* embryos, primarily focusing on the mechanisms and regulation of epidermal motility during the process.

## Translational Relevance

Reepithelialization following wounding is achieved by movement of epidermal cells across the wound site until it is covered. The mechanisms by which cells move and the signaling pathways that control their movement are well conserved throughout all multicellular organisms, meaning that studies in comparatively simple model organisms such as *Drosophila* can inform our understanding of reepithelialization in humans.

## Clinical Relevance

Prior to completion of reepithelialization, wounds are at risk of infection. In circumstances where reepithelialization is slow or fails completely, such as in chronic wounds, this risk is greatly increased. The development of therapies to accelerate reepithelialization, or reactivate it when it has failed completely, would therefore be an important clinical advance. Enhancement of reepithelialization could also reduce the need for skin grafts for large wounds. Studying reepithelialization in simple model organisms is improving our understanding of the process at the molecular level. This knowledge will aid the development of novel therapies to enhance the reepithelialization.

## Discussion of Findings and Relevant Literature

The epidermis is an epithelium whose primary function is to act as a barrier against toxins and microorganisms, but is also essential to prevent fluid loss from the body.^1^ This barrier function is lost when the epidermis is damaged, so its rapid and complete repair is a vital element of wound healing. The development of therapies that significantly accelerate reepithelialization would be an enormous clinical advance. Reepithelialization following wounding occurs by migration of epidermal cells from the surrounding intact tissue into the wound, until the advancing epidermal edges meet and fuse, thus restoring epidermal integrity.^2^ Following wounding, epidermal cells around the wound margin switch from their normal static state to a motile state, and this enables them to begin their migration into the wound.^3^ One of the key changes in this switch to a motile state is a substantial reorganization of the cell's actin cytoskeleton. The actin cytoskeleton is a network of filaments within the cell and dynamic rearrangement of this network is the main driver of cell migration during reepithelialization.^3^ To understand how reepithelialization is achieved, it is therefore necessary to understand how the actin cytoskeleton is regulated in the epidermis during the process. This can be investigated using cell culture models,^4^ but these do not accurately reproduce the complex environment found within wounded tissue, so model organism studies are also necessary. While mammalian models provide the closest approximation to human skin, it is difficult to analyze reepithelialization at the molecular level in mammals. An attractive alternative model system for analyzing the actin cytoskeleton during reepithelialization is the *Drosophila* embryo. The epidermis of the *Drosophila* embryo is considerably simpler than that of humans, consisting of a single layered epithelium attached to a thin basement membrane.^5^ This simplicity makes the *Drosophila* embryo a useful model for exploring the fundamental mechanisms of reepithelialization. One useful feature of the *Drosophila* embryo for this work is that the process of reepithelialization can be imaged in live embryos with high spatial and temporal resolution.^6^ This allows the changes that occur in epidermal cell behavior following wounding to be observed in great detail, including changes in the actin cytoskeleton. A further useful feature of *Drosophila* for this analysis is its genetic tractability. Genes can be inserted or removed from the genome with relative ease, allowing the function of individual genes and proteins in reepithelialization to be readily assessed.^7^ In addition, it is possible to perform genome-wide genetic screens to identify novel wound-related genes.^8^

### The mechanisms that drive reepithelialization in *Drosophila* embryos

**Figure 1.**
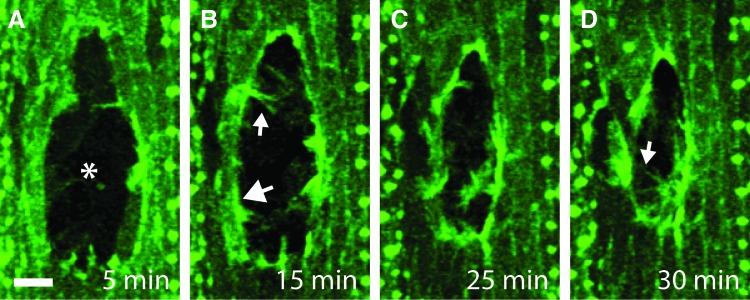
Live imaging of wound reepithelialization in the *Drosophila* embryo. The epidermis of a *Drosophila* embryo expressing a fluorescent marker of actin is wounded using a laser (wound site indicated by asterisk in panel **A**). Healing of the wound is then imaged live using a confocal microscope. **(A)** 5 min after wounding actin begins to accumulate at the wound edge. **(B)** After 15 min an actomyosin cable has formed around most of the wound edge (indicated by large arrow) and actin protrusions project into the wound (indicated by small arrow). **(C)** Contraction of the actomyosin cable reduces the wound area. **(D)** Finally, actin protrusions join the opposing wound edges to complete wound closure (indicated by arrow). Scale bar indicates 10 μm. Images captured at indicated time points after wounding. To see this illustration in color, the reader is referred to the web version of this article at www.liebertpub.com/wound

**Figure 2.**
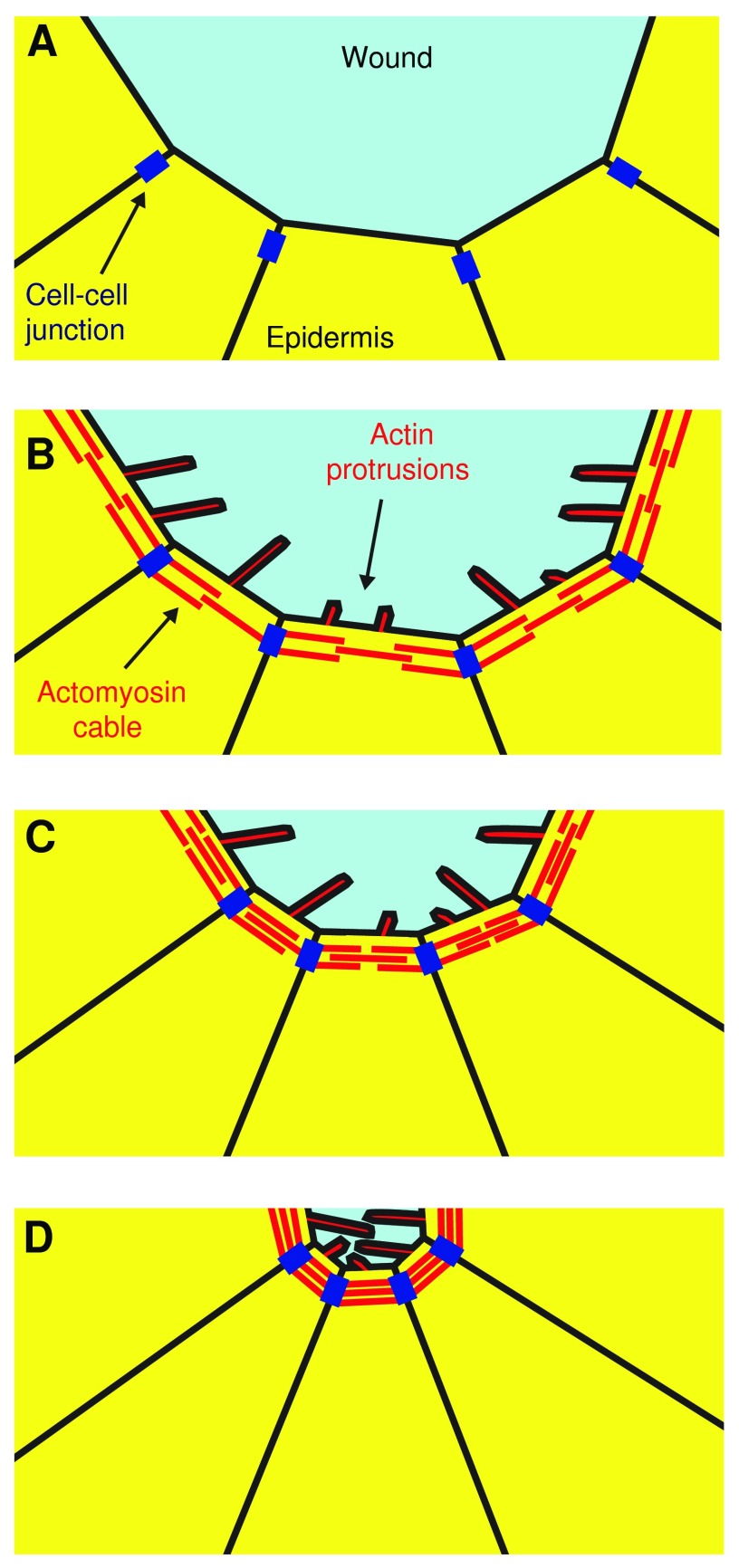
Cartoon illustrating the mechanism of wound reepithelialization in the *Drosophila* embryo. **(A)** A wound is sustained on the epidermis of a *Drosophila* embryo. **(B)** After ∼15 min, an actomyosin cable is formed along the wound edge, linked from one cell to the next by cell–cell adhesions. Actin protrusions also form and project into the wound. **(C)** Contraction of the actomyosin cable then reduces the wound area. The duration of wound contraction can vary from 15 min to several hours depending on the size of the wound. **(D)** When wound area is sufficiently small, actin protrusions on opposing edges of the wound interdigitate to join the wound edges and complete closure. To see this illustration in color, the reader is referred to the web version of this article at www.liebertpub.com/wound

Live imaging of *Drosophila* embryos expressing fluorescently tagged markers of the actin cytoskeleton has been widely used to investigate the mechanisms by which reepithelialization of the embryonic epidermis occurs.^6,9–11^ In these studies, the epidermis is wounded using a needle or laser and then closure of the wound is live imaged using a confocal microscope (Fig. 1). This research has precisely revealed how the actin cytoskeleton drives reepithelialization in this system. The early stages of wound closure are dependent on a structure called an actomyosin cable, which consists of a bundle of actin filaments cross-linked together by the motor protein Myosin-II to form a contractile cable.^6^ Following wounding, an actomyosin cable rapidly assembles around the circumference of the wound, linked from one cell to the next via cell–cell junctions (Figs. 1 and 2). Once assembled, the actomyosin cable gradually constricts, which results in a gradual reduction in the size of the wound.^6^ The action of the actomyosin cable has been likened to that of a purse string and consequently this mode of wound closure is known as purse-string wound healing.^12^ Following contraction of the wound by the actomyosin cable, a second actin-dependent process then takes over to complete wound closure. This final step of reepithelialization is mediated by actin protrusions, which are dynamic structures formed on the surface of the cells surrounding the wound.^6,9^ These protrusions are formed as a result of the rapid assembly of actin filaments immediately beneath the plasma membrane. Two types of actin protrusion are observed at wound edges; filopodia, which are thin needle-like projections, and lamellipodia, which are broad, sheet-like projections. These dynamic protrusions allow the cells on opposite sides of the wound to “search” for one another and provide the first points of contact between the opposing wound edges, thus initiating the final step of closure whereby the epidermis is resealed.^13^ The interdigitation of protrusions from opposing sides of the wound has been likened to the closing of a zipper and hence this process is known as “zippering”.^14^ Notably, zippering is observed when adhesions form between cultured human keratinocytes, indicating that this process is also likely to be important during human wound healing.^15^ Once the epidermis is completely resealed, the actomyosin cable and actin protrusions are dismantled and the wound-edge cells return to a nonmotile state.^6^ The duration of wound closure varies from around 30 min for very small wounds to several hours for larger wounds.^10^ While the actomyosin cable is of more importance in the early stages of wound closure and actin protrusions are more important in latter stages, there is in fact an overlap in the timescales over which these structures contribute to closure.^10^ Furthermore, if formation of either the actomyosin cable or actin protrusions is inhibited, the other structure can compensate to achieve wound closure alone, albeit with substantially reduced efficiency.^6^ This indicates that optimal wound closure is achieved using a synergistic combination of an actomyosin cable and actin protrusions, however, the system is sufficiently adaptable to function using one mechanism alone.

### Regulation of reepithelialization in *Drosophila* embryos

**Figure 3.**
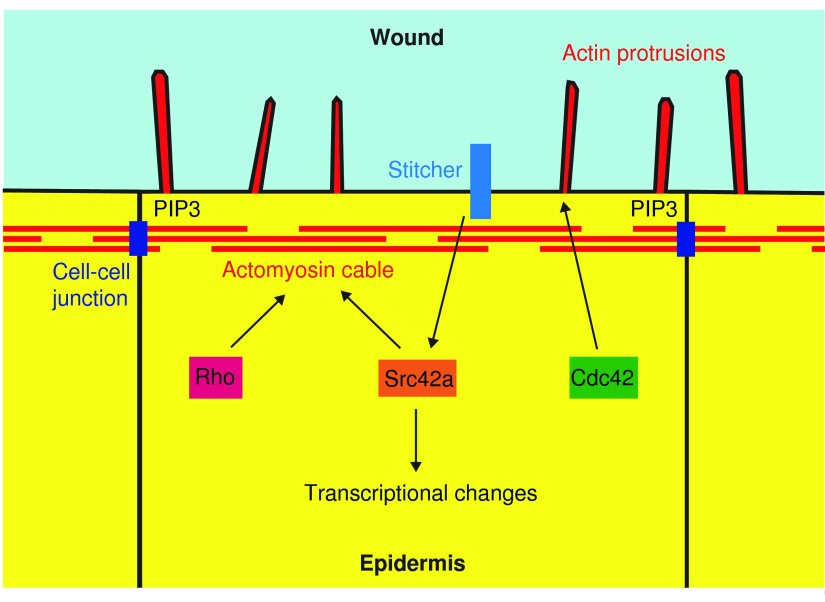
Signaling mechanisms regulating actin assembly during reepithelialization in *Drosophila* embryos. Figure shows the signaling mechanisms that operate in cells at the edges of epidermal wounds in *Drosophila* embryos to control the formation of an actomyosin cable and actin protrusions. To see this illustration in color, the reader is referred to the web version of this article at www.liebertpub.com/wound

As discussed above, reepithelialization in *Drosophila* embryos is primarily driven by the actin cytoskeleton, so the mechanisms that regulate actin are of great importance in controlling the initiation and progression of wound closure (Fig. 3). As in many other situations, the Rho family of small GTPases play key roles in regulating actin during reepithelialization in *Drosophila* embryos.^6^ The Rho GTPases are a group of closely related proteins that are activated in response to extracellular signals.^16^ When activated, Rho GTPases can bind to and regulate a suite of different proteins. Each member of the Rho family regulates a different set of proteins and therefore triggers different changes in the cell when activated. Two members of the Rho family are known to have key roles in regulating actin during reepithelialization in *Drosophila* embryos.^6^ The first of these is the founding member of the family, Rho. This GTPase is important in regulating the formation of contractile actin structures, and in embryos in which the gene encoding Rho is mutated, the actomyosin cable fails to form at wound edges, revealing Rho to be a crucial regulator of cable assembly.^6^ The mechanism by which Rho promotes actomyosin cable formation during wound healing has not been studied in detail, but is likely to involve its two key effectors; Rho-associated protein kinase (ROCK), which regulate the binding of Myosin-II to actin filaments; and Diaphanous, which regulates actin filament nucleation and elongation.^16^ Both of these Rho effectors are important in actomyosin cable assembly during wound healing in *Drosophila* pupae.^17^ The other Rho family member known to be important for reepithelialization is Cdc42. Absence of this GTPase results in a failure to form actin protrusions along the wound edge, indicating that Cdc42 promotes protrusion formation.^6^ Rho and Cdc42 are therefore key regulators of actin during reepithelialization and it is assumed that they are rapidly activated in wound edge cells when the epidermis is damaged, however, the mechanisms triggering this activation are not yet understood.

Another protein pivotal to reepithelialization is the receptor tyrosine kinase Stitcher. Loss of Stitcher results in failure of wound closure, indicating a key role for this protein in triggering wound responses.^18^ Stitcher promotes wound closure both by inducing changes in gene transcription and by activating actomyosin cable assembly. In this review, we will only discuss the transcription-independent roles of Stitcher in wound healing. The formation of an actomyosin cable in response to Stitcher activation requires the recruitment of the protein kinase Src42a to the intracellular domain of Stitcher.^19^ The mechanism by which Src42a then promotes actin assembly is currently unknown. In other systems, Src42a regulates proteins within cell–cell adhesions^20,21^ and an interesting possibility is that Src42a promotes actomoysin cable formation by inducing changes at the cell–cell adhesions around the wound to which the cable is attached.^22^ Notably, changes in the composition of these cell–cell adhesions has been shown to be important in triggering actin assembly at the wound edge.^9^ Loss of the scaffolding protein Par3 from these adhesions leads to the accumulation of the lipid phosphatidylinositol 3,4,5-trisphosphate (PIP3) at the wound edge and this triggers the formation of actin protrusions.^9^

Further signals that may be important in controlling the actin cytoskeleton during wound healing include Ca^2+^ ions and hydrogen peroxide (H_2_O_2_). Ca^2+^ levels become elevated in cells surrounding the wound immediately after the epidermis has been damaged and this leads to the release of H_2_O_2_ from these cells due to the activation of the H_2_O_2_-generating enzyme Dual oxidase.^23^ The importance of Ca^2+^ and H_2_O_2_ for reepithelialization in embryos is not clear, although it is known that the H_2_O_2_ released by wound edge cells attracts macrophages to the wound and that Ca^2+^ regulates reepithelialization in *Drosophila* pupae.^17,23,24^

While many regulators of *Drosophila* reepithelialization have been identified, the initial trigger that sets the process in motion has so far remained elusive. One possibility is that this initial trigger is mechanical. Wounding of the epidermis will lead to changes in the mechanical forces experienced by the cells surrounding the wound and this could be sensed by mechanosensitive proteins such as ion channels.^25^ Notably, the Ca^2+^ rise observed in the *Drosophila* embryonic epidermis following wounding is dependent on the Transient Receptor Potential M Ca^2+^ (TRPM) channel, the mammalian homolog of which is known to be mechanosensitive.^23,26^ Another possible trigger of reepithelialization is a diffusible molecule released when a wound is sustained, perhaps from damaged epidermal cells or from the underlying tissue. In mammals, diffusible molecules released from the wound site, either by cell rupture or by active secretion, are known to signal the existence of the wound to surrounding cells.^27^ In *Drosophila* larvae, breach of the epidermal basal lamina by wounding enables the blood-borne growth factor Pvf1 to bind to, and activate, a receptor in the epidermis and this triggers wound-edge epidermal cells to extend actin protrusions into the wound.^28^ It remains to be elucidated whether similar mechanisms also operate in *Drosophila* embryos, but it is possible that one or more such wound-released factors might be the hitherto unidentified ligand(s) of Stitcher.

### Similarities and differences between reepithelialization in *Drosophila* embryos and human skin

Reepithelialization in *Drosophila* embryos has similarities with the process in humans, but also differences. Where features of the process are similar between the two species it is more likely that knowledge gained in *Drosophila* will be directly translatable to humans. If a feature is different, the knowledge may be less useful, although these differences may be instructive, for example it may help us understand why initiation of reepithelialization is comparatively slow in humans. As in *Drosophila*, reepithelialization is believed to be an actin driven process in human skin and much of the regulatory machinery controlling the actin cytoskeleton is conserved between the two species, meaning that knowledge of actin regulation in *Drosophila* is likely to be useful in understanding the equivalent processes in human skin.^3^ For example, as in *Drosophila* embryos, Rho GTPases and receptor tyrosine kinases are important in regulating reepithelialization in mammals.^29,30^ A notable difference between *Drosophila* embryos and human skin is that in adult humans, reepithelialization does not appear to involve the purse-string mechanism discussed in 4.1. Instead, movement of the epidermis across the wound is believed to occur by crawling of keratinocytes across the wound matrix using actin protrusions.^3^ While actin protrusions also contribute to reepithelialization in *Drosophila* embryos (and are probably regulated by related proteins) their role is largely confined to the late stages of wound closure.^6,10^ Interestingly, this difference in wound closure mechanism may be due to the difference in developmental stage, rather than the difference in species. Embryos from a diverse range of species including mice and chicken reepithelize skin wounds using actomyosin purse strings, while their adult counterparts do not.^3^ Since embryonic wounds heal without leaving scars in a variety of organisms, there has been considerable interest in understanding the differences between embryonic and adult wound healing.^31^ Unlike *Drosophila* embryos, *Drosophila* larvae reepithelialize without using purse strings; so, comparison of embryonic and larval healing in *Drosophila* may provide a useful means of investigating how and why organisms switch to using a different mechanism to close wounds after embryonic development is complete.^32^

### Parallels between reepithelialization and morphogenesis in *Drosophila* embryos

**Figure 4.**
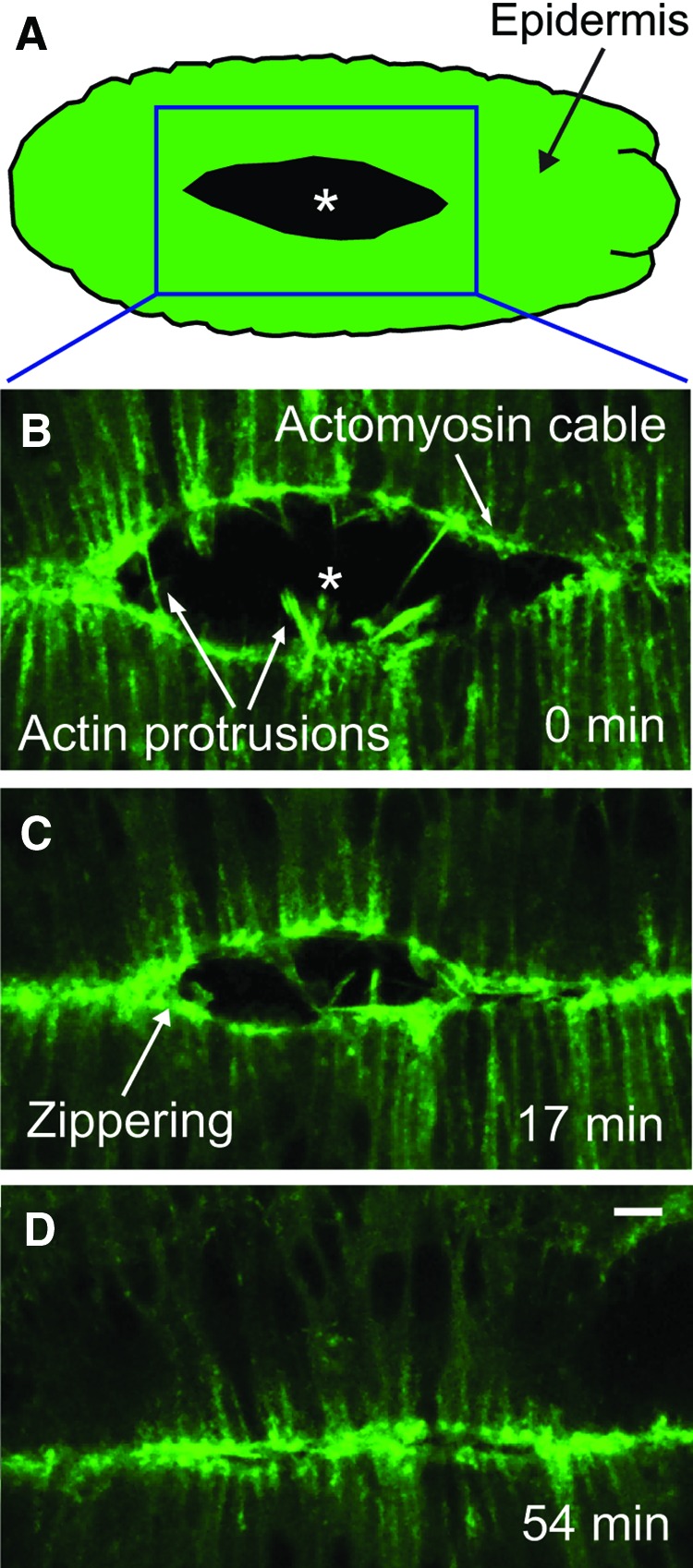
Live imaging of dorsal closure, a morphogenetic process similar to reepithelialization. During dorsal closure a large hole in the developing epidermis (indicated by asterisk in panels **A** and **B**) is gradually closed. **(A)** The position of the epidermal hole on the dorsal surface of the embryo. **(B–D)** A time course of the latter stages of dorsal closure in a live embryo expressing fluorescently tagged actin. **(B)** Mid dorsal closure. **(C)** Late dorsal closure. **(D)** Completion of dorsal closure. Scale bar indicates 10 μm. To see this illustration in color, the reader is referred to the web version of this article at www.liebertpub.com/wound

During embryonic development, the tissues that make up an organism are constructed in a series of events collectively known as morphogenesis. It is relatively common for these morphogenetic events to involve a step in which two epithelial edges move toward one another and then fuse to form one continuous epithelium, a well-known example being neural tube closure in vertebrates.^33,34^ An obvious parallel can be drawn between such processes and reepithelialization, which also involves the movement and fusion of epithelial edges. Research carried out using *Drosophila* embryos has revealed that this parallel is more than just superficial and that there are in fact striking similarities between wound healing and epithelial closures that occur during tissue morphogenesis.^6,34^ Dorsal closure is a morphogenetic event that occurs about halfway through *Drosophila* embryogenesis in which a large hole in the developing epidermis is closed.^35^ The hole is closed by the movement of two sheets of epidermal cells toward one another until the leading edges of the two sheets meet and fuse (Fig. 4). The cells at the leading edges of the two epithelial sheets play a key role in driving the closure process. As during wound healing in *Drosophila* embryos, these cells form an actomyosin cable, linked from one cell to the next by cell–cell junctions.^36^ Contraction of this cable provides a force that helps drive closure of the hole in a manner similar to that observed during purse-string wound healing. The dorsal closure leading edge cells also form actin protrusions and, as during wound healing, these protrusions zipper the two epidermal edges together when they come into contact.^13,37,38^ As well as using the same actin structures to drive epithelial closure, it appears that the regulatory mechanisms that control actin assembly are very similar during dorsal closure and wound healing, for example the roles of the Rho GTPases and PIP3 are conserved.^6,9^ The tissue that underlies the dorsal hole, which is called the amnioserosa, undergoes contraction during dorsal closure and this aids closure by pulling the advancing epidermal edges toward one another.^39^ This is similar to wound healing in human skin, in which contraction of the underlying wound matrix draws the epidermal edges closer together, thus aiding reepithelialization.^2^ Morphogenetic epithelial closure events in other organisms including mice have been shown to occur by similar mechanisms to dorsal closure and hence also share similarities to wound healing.^33^ This opens up the possibility of using our knowledge of tissue morphogenesis to inform our understanding of wound healing and vice versa.

## Summary

The reepithelialization phase of wound healing is complex and poorly understood. Our efforts to understand it more fully have made use of a wide variety of model organisms and it is through integrating the knowledge gained in each of these organisms that a comprehensive understanding of the process will emerge. The *Drosophila* embryo has been useful in improving our understanding of reepithelialization at the molecular level, in particular the role played by the actin cytoskeleton in driving reepithelialization, and the signaling mechanisms that control actin assembly during the process. In a comparatively short period of time, studies in *Drosophila* have identified an extensive list of proteins that function during reepithelialization of the embryonic epidermis. Most of these proteins are evolutionarily conserved and it is important that we now establish whether these proteins perform similar roles in humans or mammalian model organisms. An important realization that has come from studying reepithelialization in *Drosophila* embryos is the similarity between the morphogenesis of epithelial tissues and their repair following wounding. This knowledge may lead to new approaches in the quest to develop novel therapies to improve wound healing.

Take-Home Messages

• The *Drosophila* embryo provides a simple model system for analyzing the evolutionarily conserved molecular and cellular mechanisms that underlie the reepithelialization of wounds. The main advantages of the *Drosophila* embryo for this analysis is the ability to live image reepithelialization with high resolution and the ability to genetically manipulate the embryos and thereby investigate the role of individual genes and proteins in the process.
• Numerous cellular signals involved in triggering and regulating reepithelialization in *Drosophila* embryos have been identified and analyzed in recent years. Many of these signals are likely to perform similar functions in human reepithelialization and are therefore potential therapeutic targets.
• Studies using *Drosophila* embryos have shown that the mechanisms by which wounds are reepithelialized are surprisingly similar to processes that occur when epithelial tissues are first formed during embryonic development. This opens up the possibility of using our existing knowledge of embryology to improve our understanding of wound healing.

## About the Authors

**Tom H. Millard, PhD,** and **Yutaka Matsubayashi** are members of the Healing Foundation Centre at the University of Manchester and carry out research that focuses on the molecular mechanisms of wound healing, primarily using *Drosophila* as a model system.

## Abbreviations and Acronyms

H_2_O_2_: hydrogen peroxide
PIP3: phosphatidylinositol 3,4,5-trisphosphate
ROCK: Rho-associated protein kinase
TRPM: transient receptor potential M Ca^2+^
